# A population-based retrospective study of the modifying effect of urban blue space on the impact of socioeconomic deprivation on mental health, 2009–2018

**DOI:** 10.1038/s41598-022-17089-z

**Published:** 2022-07-29

**Authors:** Michail Georgiou, Zoë Tieges, Gordon Morison, Niamh Smith, Sebastien Chastin

**Affiliations:** 1 School of Health and Life Sciences, Glasgow Caledonian University, 70 Cowcaddens Road, Glasgow, Scotland G4 0BA UK; 2 Geriatric Medicine, Usher Institute, University of Edinburgh, 51 Little France Crescent, Edinburgh, Scotland EH16 4SA UK; 3 SMART Technology Centre, School of Computing, Engineering and Built Environment, Glasgow Caledonian University, 70 Cowcaddens Road, Glasgow, Scotland G4 0BA UK; 4 School of Computing, Engineering and Built Environment, Glasgow Caledonian University, 70 Cowcaddens Road, Glasgow, Scotland G4 0BA UK; 5 Department of Movement and Sports, Ghent University, Watersportlaan 2, 9000 Ghent, Belgium

**Keywords:** Psychiatric disorders, Environmental sciences, Environmental social sciences, Diseases, Health care

## Abstract

The incidence of mental health disorders in urban areas is increasing and there is a growing interest in using urban blue spaces (urban waterways, canals, lakes, ponds, coasts, etc.) as a tool to manage and mitigate mental health inequalities in the population. However, there is a dearth of longitudinal evidence of the mechanisms and impact of blue spaces on clinical markers of mental health to support and inform such interventions. We conducted a 10-year retrospective study, following STROBE guidelines, using routinely collected population primary care health data within the National Health Service (NHS) administrative area of Greater Glasgow and Clyde for the North of Glasgow city area. We explored whether living near blue space modifies the negative effect of socio-economic deprivation on mental health during the regeneration of an urban blue space (canal) from complete dereliction and closure. A total of 132,788 people (65,351 female) fulfilling the inclusion criteria were entered in the analysis. We established a base model estimating the effect of deprivation on the risk of mental health disorders using a Cox proportional hazards model, adjusted for age, sex and pre-existing comorbidities. We then investigated the modifying effect of living near blue space by computing a second model which included distance to blue space as an additional predicting variable and compared the results to the base model. Living near blue space modified the risk of mental health disorders deriving from socio-economic deprivation by 6% (hazard ratio 2.48, 95% confidence interval 2.39–2.57) for those living in the most deprived tertile (T1) and by 4% (hazard ratio 1.66, 95% confidence interval 1.60–1.72) for those in the medium deprivation tertile (T2). Our findings support the notion that living near blue space could play an important role in reducing the burden of mental health inequalities in urban populations.

## Introduction

The global burden of diseases due to mental health disorders is increasing^1^. The World Health Οrganisation forecasts that mental health disorders will be the leading cause of Disability Adjusted Life Years in middle to high-income countries by 2030^2^. It is estimated that 11–18% of the world’s population is affected by mental health issues^3^. However, the incidence of mental health disorders is not evenly distributed in the population, and evidence shows a strong socio-economic gradient in mental health^4–6^. The risk of mental health disorders is substantially higher for people living in economically disadvantaged areas and areas with chronic low employment rates^7–10^.

The situation is compounded by a widening “mental health treatment gap” where, worldwide, more than two-thirds of people with mental health disorders cannot access mental health services^11^. Therefore, preventive measures should be prioritised to close this gap and decrease the incidence of mental health disorders^12^. In particular, there is growing advocacy for tackling upstream determinants, the “causes of causes” of mental health disorders and health inequalities^13^, based on mounting evidence of efficacy^14^, and added social and economic societal benefits^15,16^.

Urbanisation, considered one of the “causes of causes”, is an important risk factor for mental health and health inequalities^17^. As the world is becoming increasingly urban, there is growing interest in using the built environment to mitigate some of the adverse effects of urbanisation on mental health and positively promote good mental health and prevent disease^18^. Urban green spaces have long been seen as important assets for health promotion^19^; they are a common setting for social prescription^20^. More recently, it has emerged that water environments within an urban setting also have promising mental health enhancing capacities. Some epidemiological studies have shown that living near urban coastline, rivers, canals and lakes, collectively defined as “blue spaces”, is associated with lower psychological distress and better self-reported mental health^21,22^. Moreover, recent cross-sectional evidence suggests that this effect is only seen amongst populations in the lowest socio-economic stratum, indicating that living near a blue space may mitigate inequality in mental health^23^.

This study aimed to investigate whether living near blue space longitudinally modifies the effect of socio-economic deprivation on mental health. Hence, we studied, longitudinally, the impact of a large-scale regeneration of the Glasgow Branch of the Forth and Clyde Canal, an urban blue space, on mental health using routinely collected clinical data. The study took place during a period of significant regeneration, during which the derelict canal was transformed into an accessible waterway with a usable towpath. The area surrounding the canal experiences high levels of socio-economic deprivation, as defined by the Scottish Index of Multiple Deprivation (SIMD)^24^.

## Methods

### Ethical considerations

The study obtained approval from Safe Haven review and Local Privacy Advisory Committee (reference: GSH/19/SS/002); the NHS Greater Glasgow and Clyde Safe Haven operates under delegated ethical approving powers from the West of Scotland Research Ethics Committee. Caldicott approval for re-use of data was in place. The study adhered to strict information governance and security protocols. Databases were de-identified within the Safe Haven before being securely transferred to a secure workspace in the Aridhia digital research platform (AnalytiXagility). Data access was restricted to three named researchers (ZT, MG and SC).

### Study design and setting

**Figure 1:**
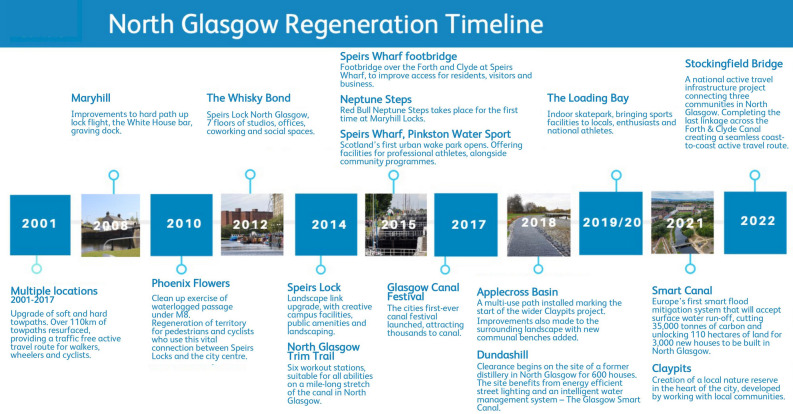
Schematic diagram showing the timeline of the canal regeneration programme in the North of Glasgow City from complete closure and dereliction to re-opening in North Glasgow in Scotland^10^.

**Figure 2:**
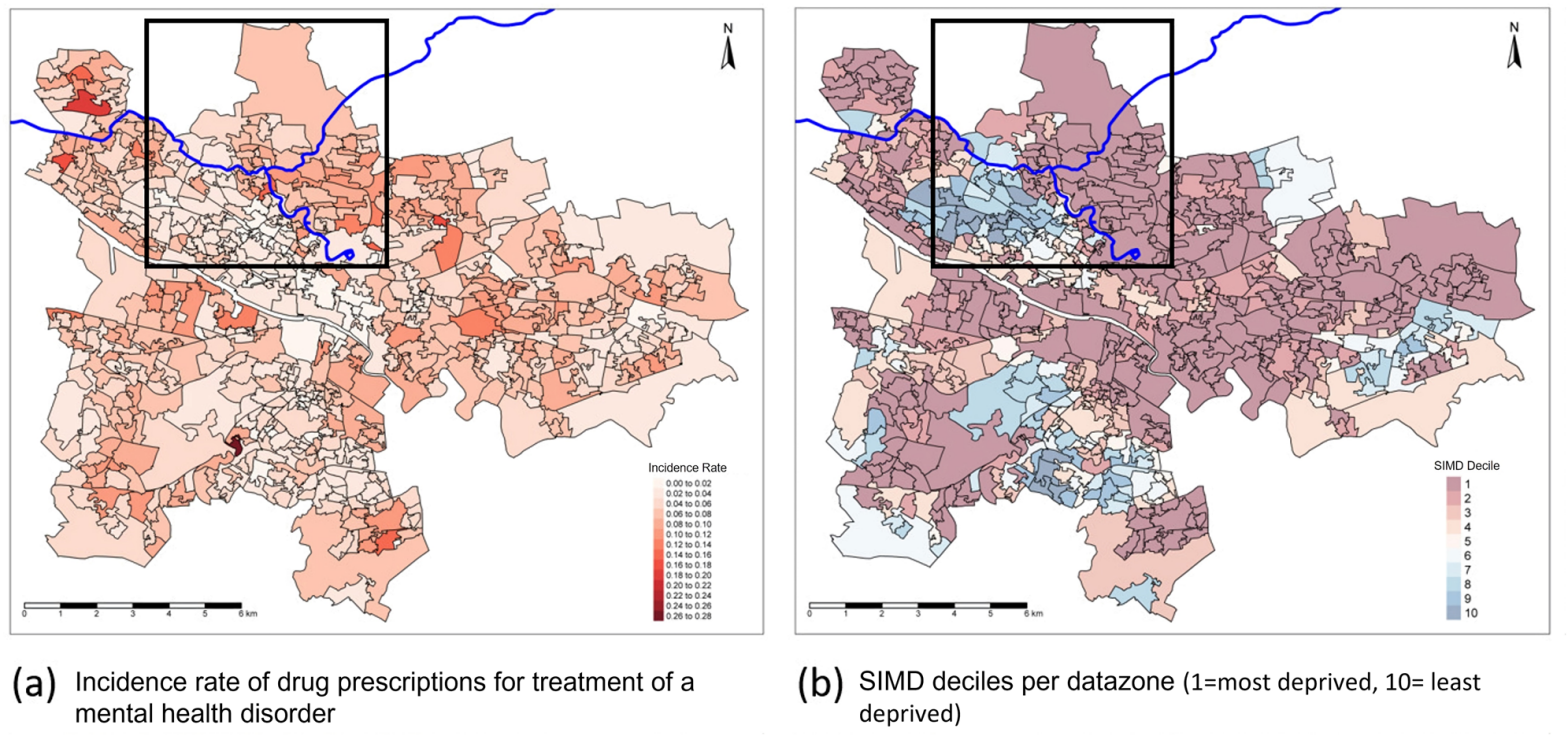
(**a**) Map of the incidence of drug prescriptions for treatment of a mental health disorder (number of people prescribed a drug for treatment of a mental health disorder/datazone population) for Glasgow in 2009 (starting date of analysis); (**b**) Map of Scottish Index of Multiple Deprivation (SIMD) 2004 deciles (1 = most deprived, 10 = least deprived) for each datazone in Glasgow. Blue line represents the canal in both maps. Study area within rectangle shape. *Maps were generated using R software version 3.6.1.^29^ URL: https://cran.r-project.org/bin/windows/base/old/3.6.1/.

We conducted a population-based retrospective cohort study, following the STROBE guidelines^25^, of the impact of the regeneration of the North Glasgow branch of the Forth and Clyde Canal on the mental health of the population of North Glasgow, Scotland, UK. Rather than a “before-after” regeneration design, this study entailed a survival analysis during a period of ongoing regeneration events. This is thought to be a powerful approach in public health research for identification of risk and prognostic factors^26^. A schematic diagram showing the canal’s regeneration is displayed in Fig. 1. The canal restoration project in this area is a unique natural experiment to investigate the impact of urban blue spaces on mental health for two reasons: it is a notable example of climate change mitigation and is significant due to the surrounding socio-economic deprivation. The canal was closed entirely and left to dereliction for over 40 years before 2000 and has since undergone the most extensive canal regeneration programme in the U.K, with the primary aim of increasing the communities’ resilience to climate change. The first canal lock was re-opened as a space for recreation in the area of interest in 2006. Glasgow’s ‘Smart Canal’ project describes the regeneration of the city’s canal network for flood prevention through rainfall absorbance, offering a successful example of a Nature-Based Solution towards climate adaptation and resilience^27^. The neighbourhoods surrounding the North Glasgow branch of the canal are characterised by clustering of environmental and socio-economic deprivation, physical and mental health challenges, significant health disparities and climate change vulnerabilities^28^. Figure 2 shows (a) the geographical distribution of mental health drug prescriptions at the beginning of our study period and (b) the geographical distribution of social deprivation near the start of the canal regeneration.

Glasgow is the most populous city in Scotland, with a population of 593,245 according to the 2011 UK Census^30^. The study setting and area are described in detail elsewhere^31^. In brief, the study area encompassed the canal network that runs through the North of Glasgow (area 33 km^2^), including the Glasgow Branch of the Forth and Clyde Canal, where most of the regeneration took place. Within the study, we used the Glasgow City boundary as the upper boundary (North) and the M8 motorway, which separates North Glasgow from the City Centre, as the lower boundary (South).

### Data sources

We linked data from the National Records of Scotland (NRS) to routinely collected population health data within the NHS administrative area of Greater Glasgow and Clyde, held within the NHS Scotland Information Services Division Safe Haven. Specifically, we used the Local Enhanced Service database from General Practice (GP) surgeries and the pharmacy prescription database. The GP Local Enhanced Services database captures patient information recorded from the Scottish Primary Care Information Resource, which provides primary care data for Scotland^32^. This database covers a range of chronic health conditions managed in primary care. In the UK, most people are registered with an NHS GP surgery^10^. The pharmacy prescription database contains prescription and dispensation details. This includes all prescribed and dispensed medications within the Greater Glasgow and Clyde area from 2008 to 2019.

NHS Greater Glasgow and Clyde Safe Haven conducted the data linkage based on the Community Health Index (CHI) number, a single patient identifier throughout NHS Scotland. We subsequently linked the datazones where a person resided to socio-economic deprivation status according to the Scottish Index of Multiple Deprivation (SIMD)^33^.

### Independent variable: prescription of psychotropic medications

We estimated the diagnosis of mental health disorders through clinical records of mental health drug prescriptions. We used GP and pharmacy prescription databases to establish the date an individual was prescribed a drug for the treatment of a mental health disorder between 2009 and 2018. We considered psychotropic drugs for the treatment of mental health disorders. The full list is provided in Supplementary Table S1 in the supplementary material.

The GP Local Enhanced Services database provides data on clinical events, which refer to any form of diagnosis, measurement, reading, prescription, medication or other clinical finding recorded in the patient’s medical history. These data include dates of clinical events, Read Codes, descriptions for each event, and the postcode sector for GP practices.

Read Codes are a national standard coded thesaurus of clinical terms for coding and recording all relevant information from a patient encounter^34^. Read Codes were developed and maintained by the Health and Social Care Information Centre based in Leeds, UK. They are used by GPs throughout the UK NHS. GP practices in Scotland use 5-byte Version 2 Read Codes which are based on International Classification of Diseases (ICD-9-CM) diagnosis and procedure codes from the World Health Organization^35^.

The pharmacy database provides data on mental health drug prescription events and dates as part of the routine GP population health data within the NHS administrative area of Greater Glasgow and Clyde, held within the NHS Scotland Information Services Division Safe Haven. The starting date of the prescriptions dataset was 01 January 2008.

### Distance to blue space

We calculated how far people live from a blue space as the Euclidean distance (i.e. straight-line) between population-weighted centroids of individuals’ 2011 data zones and the nearest portion of the canal using the R package 'sf'^36^.

### Mental health determinants/confounders

#### Socio-economic deprivation

We estimated the level of socio-economic deprivation using the 2016 SIMD^33^. SIMD identifies concentrations of multiple deprivation at the small area (datazone) level across seven domains: income, employment, housing, health, education, and geographic access. The SIMD is the recommended measure for monitoring health inequalities in Scotland^37^. We ranked SIMD scores from highest to lowest for the 746 Glasgow datazones based on the 2011 Census and calculated three SIMD tertiles; tertile 1 being the most deprived and tertile 3 being the least deprived.

#### Comorbidities variable: chronic health indices

We estimated individuals’ burden of physical health comorbidity using Read Codes based on an algorithm developed by Metcalfe et al.^38^. Details of the Elixhauser Method are described elsewhere^10,38^. The authors generated code lists for both the Charlson Comorbidity Index (CCI) (17 categories) and the Elixhauser Method (31 categories), and showed that the Elixhauser codes outperformed the CCI codes in predicting hip fracture mortality^38^. Hence, we used the Elixhauser code lists in our study. We included the following health conditions in the index: Chronic pulmonary disease, renal disease, obesity, other neurological disorders, peripheral vascular disorder, diabetes, cardiovascular disease and hypertension.

#### Demographics

We obtained information on sex, date of birth and date of death (if appropriate) from the NRS records, on 01 January 2000.We calculated the age of each person on the first day of our study period (01 January 2009) from their date of birth.

### Statistical analysis

**Figure 3:**
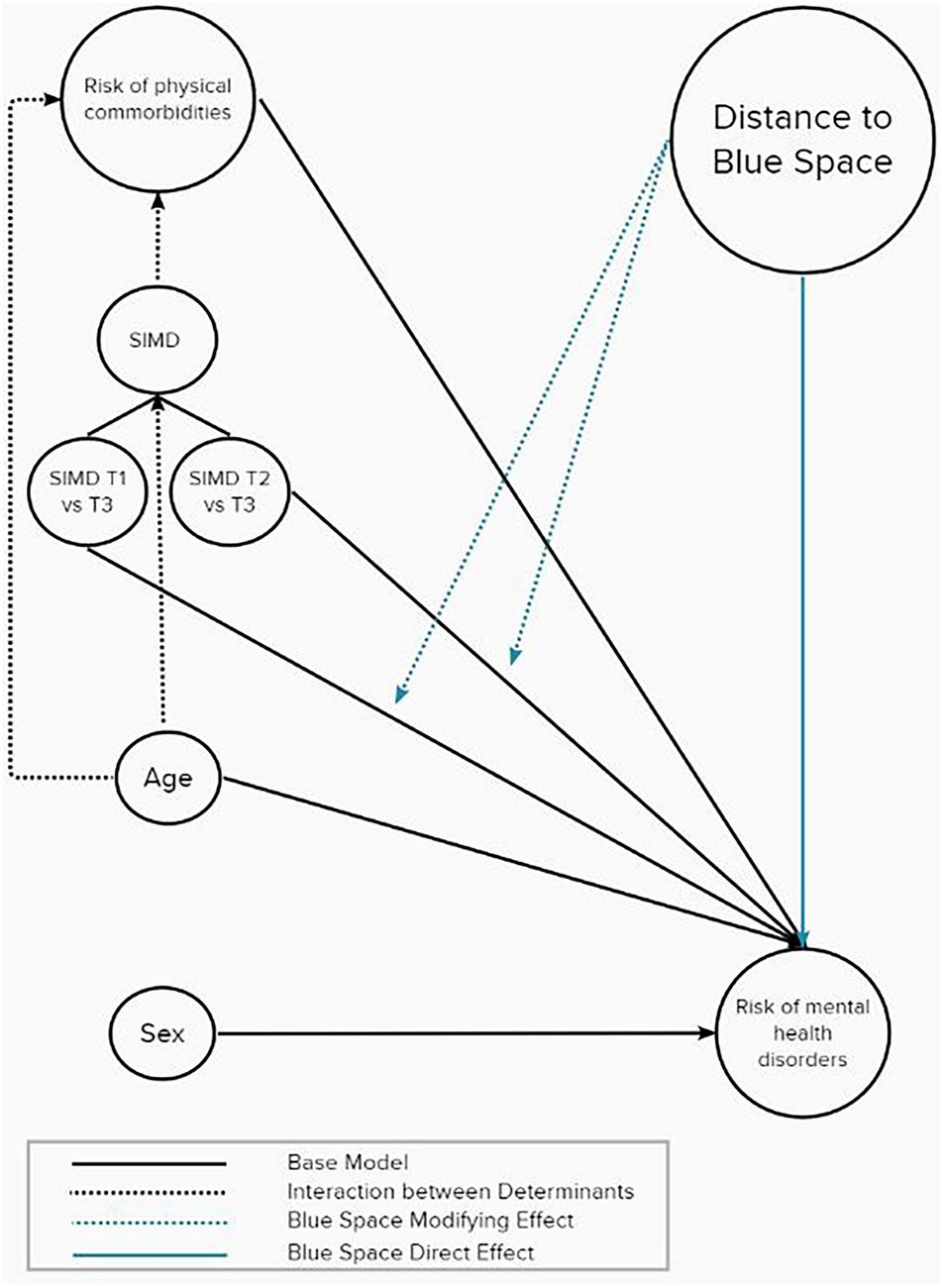
Conceptual directed acyclic graph of the associations between mental health, distance to blue space and mental health determinants/confounders; SIMD T1 (most socio-economically deprived tertile); SIMD T2 (medium socio-economic deprivation tertile).

The directed acyclic graph (DAG), presented in Fig. 3, guided our analysis. We first established a base model estimating the effect of deprivation on the risk of developing a mental health disorder using a Cox proportional hazards model^39^, with ‘time to event’ as the dependent variable taking into account the effect of common confounders; age, sex and pre-existing comorbidities^40^. We defined ‘time to event’ as the time in months to the first record of mental health drug prescription for an individual. We left-censored entry time on 1 January 2009 and right-censored follow-up time on 31 December 2018. To investigate the modifying effect of living near blue space, we computed a second model, which included distance to blue space as an additional predicting variable and compared the results to the base model. We reported Hazard Ratios (HR) and 95% Confidence Intervals (CIs) for risk and beta coefficients for effects for each model. The modifying effect of living near blue space was calculated from the difference in both effects and risks between models. We used the concordance index (C-index) to evaluate the accuracy of the prognostic ability and reliability of our models; C-index > 0.70 indicates good prognostic ability of a model^41^. We performed log-rank tests and Akaike information criterion (AIC) comparisons to test for improvement of goodness-of-fit between models. Statistical significance was tested at the 95% confidence level.

We conducted a sensitivity analysis to ascertain the robustness of the results and investigate potential reverse causation effects. We adapted our final model (with distance to blue space variable) to 14 scenarios of different cut-off ages and time periods. Specifically, we restructured our model with the distance variable (≥ 9 Ages, 2009–2018) for cut-off ages of 0, 8, 9, 10 and 18 years old for the 2009–2018, 2011–2018 and 2014–2018 time periods.

All analyses were conducted on R Version 3.6.1.^29^.

### Patient and public involvement

No patients or any members of the public were involved in the design, data analysis or conduct of this study. The study was based on deidentified historical data from Greater Glasgow and Clyde NHS Safe Haven.

## Results

### Data flow and sample characteristics

**Figure 4:**
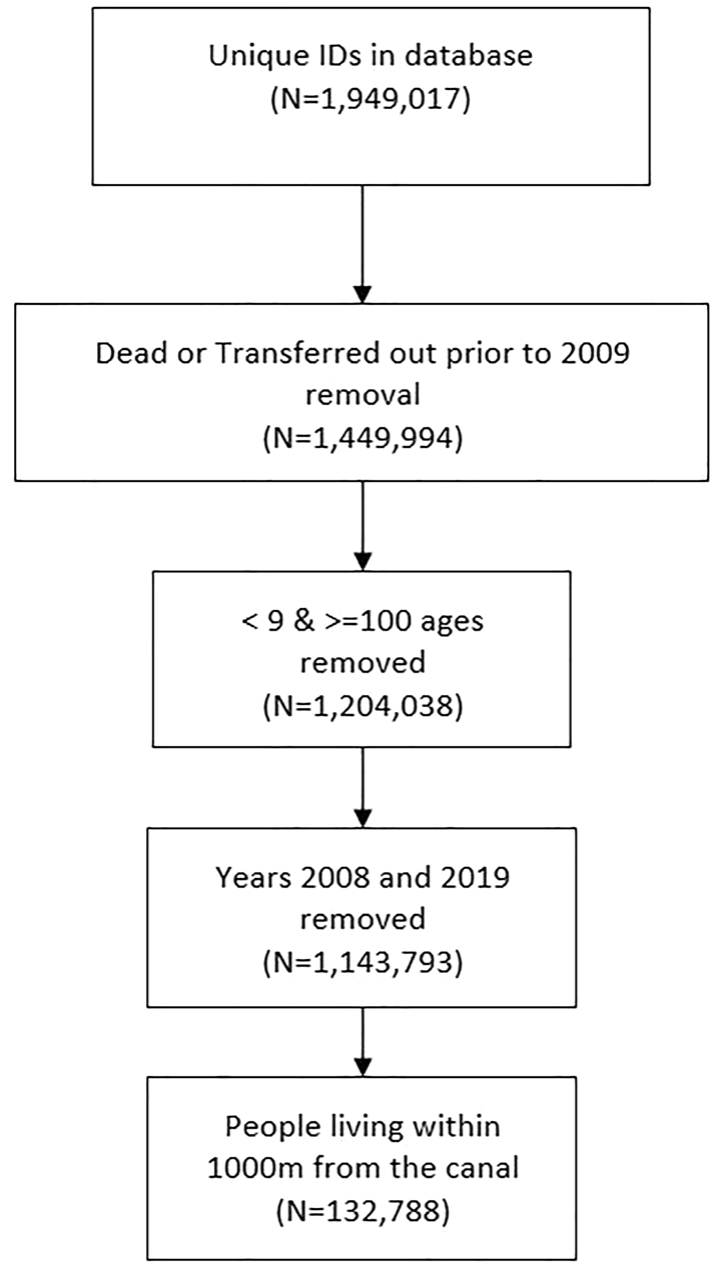
Data flow diagram.

From the 1,949,017 IDs held in the database, we excluded IDs of people who died or moved out of the area before 2009 because they were outside our study period (2009–2018). We only included IDs of people over the age of 9 in 2009 so that all individuals included would be at least 18 years old at the end of our study period (2018). This resulted in 1,143,793 IDs remaining in our dataset. Of those, 132,788 people lived within 1000 m of the canal during the study period (2009–2018). Details of the data flow and distribution of people and events are presented in Fig. 4 and Table 1, respectively.

**Table 1:** Distribution of people within analysed sample.

Overall			
	No. of people	No. of events	Ratio (events/people)
Male	67,437	8,712	12.91%
Female	65,351	11,766	18.00%
Summary	132,788	20,478	15.42%

### Base model

**Figure 5:**
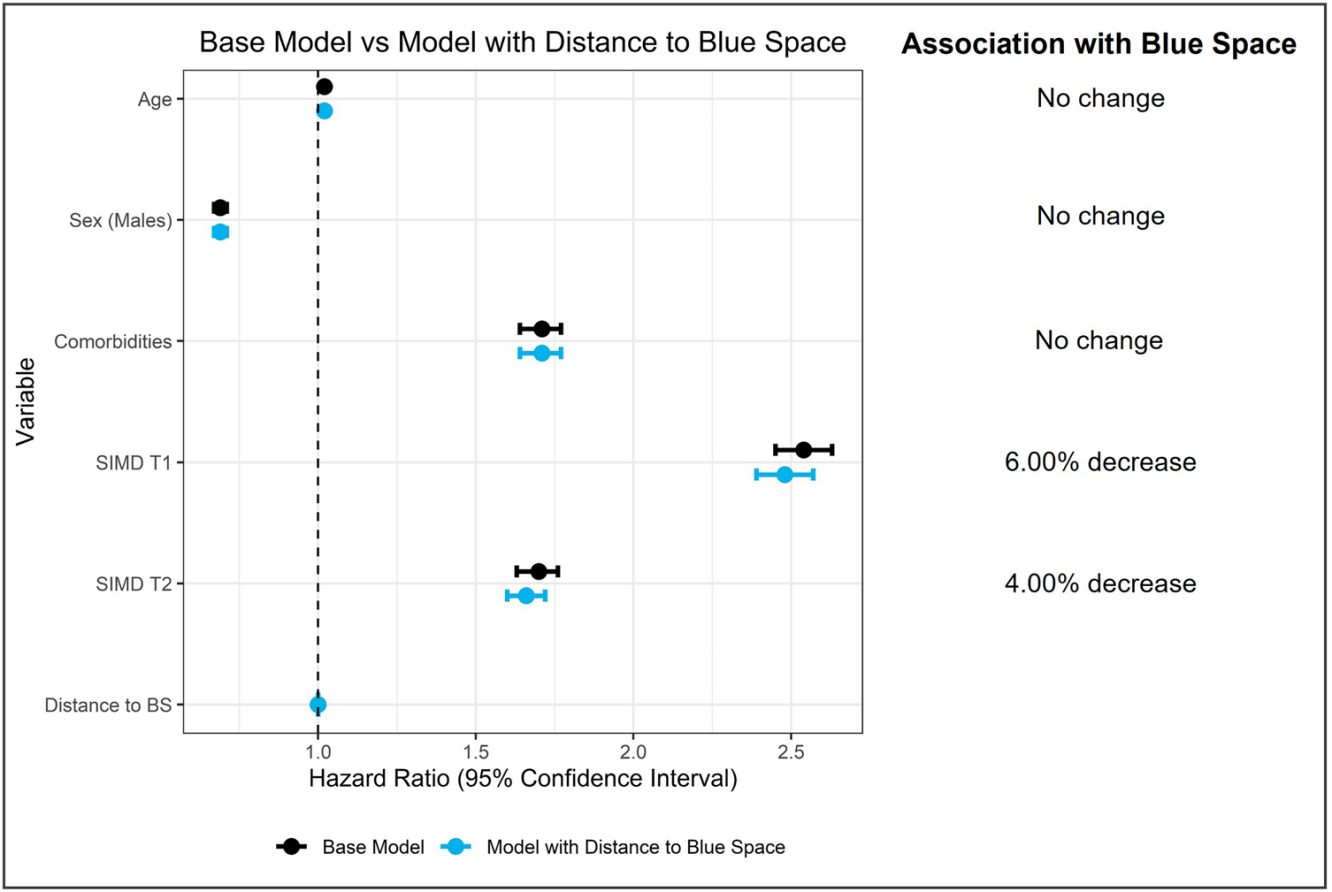
Forest plot of modifying effect of living near blue space on predictors of the risk of mental health issues.

Results of the base model are presented as forest plots in Fig. 5 below and Supplementary Table S2 in the supplementary material. The base model had a good prognostic ability of the risk of mental health disorder (C-index = 0.725, 95% confidence interval 0.721–0.728). As expected, the base model indicated a strong effect of deprivation on the risk of mental health disorders. In detail, people living in the most socio-economically deprived tertile (T1) had a 154% higher risk of mental health disorders than those living in the least deprived tertile (T3) (β = 0.931, p < 0.05; hazard ratio 2.54, 95% confidence interval 2.45–2.63) and those living in the medium deprivation tertile (T2) had a 70% higher risk than those in the least deprived tertile (T3) (β = 0.528, p < 0.05; hazard ratio 1.70, 95% confidence interval 1.63–1.76). We also observed the effects of age, sex and pre-existing comorbidities. Specifically, males had a 31% lower risk of mental health disorders than females (β = − 0.376, p < 0.05; hazard ratio 0.69, 95% confidence interval 0.67–0.71), people with pre-existing comorbidities had a 71% higher risk than those without any pre-existing comorbidity (β = 0.534, p < 0.05; hazard ratio 1.71, 95% confidence interval 1.64–1.77), and older people had a 2% higher risk than those of younger age (β = 0.021, p < 0.05; hazard ratio 1.02, 95% confidence interval 1.02–1.02).

### Modifying effect of living near blue space

The Cox proportional hazards model taking into account distance to blue space, indicated that living closer to blue space did not have a direct effect on the risk of mental health disorders (β = 0.000, p < 0.05; hazard ratio 1.00, 95% confidence interval 1.00 to 1.00) (Fig. 5 above and Supplementary Table S2 in the supplementary material). However, adding the distance to blue space variable in the model modified the negative effect of socio-economic deprivation on mental health disorders. Specifically, for people living in the most socio-economically deprived tertile (T1), the effect was attenuated by 6% (β = 0.909, p < 0.05; hazard ratio 2.48, 95% confidence interval 2.39–2.57). Similarly, for those living in the medium deprivation tertile (T2), the effect of deprivation was attenuated by 4% (β = 0.505, p < 0.05; hazard ratio 1.66, 95% confidence interval 1.60–1.72). In terms of effect size, when adding the distance to blue space variable in the model, the effect of socio-economic deprivation on mental health disorders appeared to be reduced by 2.42% for those living in the most deprived tertile (T1) and by 4.30% for those in the medium deprivation tertile (T2). As shown in Fig. 5, distance to blue space did not change the effect of age, sex or pre-existing comorbidities. Modifying effects of distance to blue space were also confirmed by statistically significant (p < 0.05) interaction terms between distance and deprivation variables. Moreover, the addition of the distance to blue space variable significantly improved our model, as indicated by a statistically significant log-rank test (p < 0.05) and lower AIC values. The good prognostic ability of the model was not affected (C-index = 0.725, 95% confidence interval 0.721–0.728).

### Sensitivity analysis

Our sensitivity analysis showed that the results were not significantly affected by changing the inclusion age or the length of the follow-up period. We present hazard ratios for our model with the distance to blue space variable in Supplementary Fig. S1, in forest plots, and Supplementary Table S3, in the supplementary material.

## Discussion

In this large longitudinal analysis of routinely collected NHS data, we investigated whether living near blue space modifies the effect of socio-economic deprivation on mental health disorders. We identified a protective modifying effect for living near blue space on the deleterious association between socio-economic deprivation and mental health disorders. Living closer to blue space attenuated the negative effect of socio-economic deprivation on mental health disorders through a 6% and 4% risk reduction both for those in the most deprived tertile (T1) and those in the medium deprivation tertile (T2), respectively (T1 vs T3: Risk from 154 to 148% higher, T2 vs T3: Risk from 70 to 66% higher). We did not find a direct effect of living near blue space on the risk of mental health disorders. Nor did we find a similar modifying effect of living near blue space on the associations between pre-existing comorbidities, sex, age and mental health disorders.

In their recent cross-sectional study, Garrett et al.^23^ reported a differential association between living near blue space and mental health. Using 12-item General Health Questionnaire (GHQ-12) scores as a mental health indicator and equivalised household income as a socio-economic status index, they found that the beneficial effect of blue space on mental health existed only for those living in the most deprived areas^23^. These findings suggest that the effect of blue space on mental health is moderated by socio-economic deprivation^23^. Our study confirms longitudinally that the beneficial effects of blue spaces are greater amongst more vulnerable and socio-economically deprived communities. Contrary to previous research, our results suggest that it is the proximity to blue space that moderates the impact of deprivation. We suggest that living near blue space may not eradicate the negative effects of socio-economic deprivation on mental health for all socio-ecomic levels, but it creates a “protective moat” effect which attenuates the increased mental health risks deriving from socio-economic deprivation. With regard to the mechanisms of these effects, a recent systematic review and meta-analysis looking at the impact of blue space on health, found that the potential mechanisms explaining the observed moderating effects may be: increased opportunities for physical activity and restoration, more social interaction and improvement of the environmental factors near blue space^42^. However, further research should be conducted to understand the actual physiological effects and how exposure to blue space may change brain physiology. In that sense, recent research looking at the effect of green spaces on brain activity has found that visual exposure to green spaces may be a health-promoting and stress-relieving experience, through adjustment of oxyhemoglobin levels^43,44^. Yet, it is still unknown whether a similar physiological mechanism applies to blue space.

Given the large size of the negative effect of socio-economic deprivation on mental health disorders (T1 vs T3: Risk from 154 to 148% higher, T2 vs T3: Risk from 70 to 66% higher), the risk attenuation by 6% and 4% for those in the most deprived (T1) and medium deprivation tertile (T2), respectively, may appear numerically small. However, we suggest that this may have an important effect at a population level in an urban environment, if blue space interventions can be developed at scale^45^.

Interestingly, the moderating effect of socio-economic deprivation has been recently also reported in a study investigating the association between blue space exposure and physical health^10^. In detail, using SIMD index as a measure of socio-economic deprivation over an 18-year period, higher exposure to blue space was found to significantly reduce the risk of physical health-related diseases, such as incident cardiovascular disease, hypertension, stroke and obesity, but only for those living in the most socio-economically deprived areas^10^. This, implies that living near blue space also moderates the risk of having pre-existing comorbidities which in turn lowers the risk of mental health disorders. We therefore suggest that, taken together, there are two main pathways of the effect of blue space on mental health; (a) through the attenuation of the negative effect of socio-economic deprivation and (b) through the direct effect of blue space on the risk of developing comorbidities, leading to lower risk of having pre-existing comorbidities and in turn mental health disorders.

With respect to the direct effect of blue space on mental health disorders, previous studies have reported a beneficial effect of closer proximity to blue space on mental health. In more detail, a recent systematic review and meta-analysis of 25 studies on the impact of urban blue spaces on human health found that people living closer to blue space had better self-reported mental health and wellbeing (Cohen’s d − 0.25, 95% confidence interval − 0.44 to − 0.07)^46^. However, the evidence base solely consisted of cross-sectional studies using mainly self-reported measures of mental health and wellbeing^46^. The lack of evidence of a direct effect of blue space on mental health disorders in our large longitudinal study of routine NHS data suggests that more longitudinal research should be conducted on the matter, using clinical markers of mental health, for conclusive evidence.

In that sense, a recent longitudinal study investigating the direct effect of growing up in areas with blue space on any subsequent psychiatric disorder in Denmark, by comparing 3000 m × 3000 m areas mainly covered by blue space to a mainly urban 3000 m × 3000 m space, found a direct protective effect for greater exposure to blue space (18% lower risk)^47^. Although these results may appear somewhat incompatible with our findings, the discrepancy could be attributed to the difference in the exposure and reference in the two studies. In our study, we used Euclidean distances from population-weighted centroids of individuals’ 2011 data zones to measure distance to blue space, while Engemann et al. used the difference in amount of blue space within a large area^47^. We would argue that our study better reflects blue space exposure at a more close-up scale relevant to nature-based therapy, but the study by Engemann et al. may better reflect the impact of the living environment^47^. As blue spaces are often narrow linear features by nature, distance metrics are generally preferred to spatial coverage metrics in research^48^. Additionally, we adjusted our models for pre-existing comorbidities, which may play a significant role in the development of mental health disorders^49^. Taken together, the absence of a pre-existing comorbidities covariate and the nature of the measures of exposure to blue space in the study by Engemann et al.^47^, may have led to overestimation of the direct rather than indirect effects of blue space on mental health.

The results of our study have several important implications. Firstly, findings confirm the modifying role of blue space on the association between socio-economic deprivation and mental health. Combined with the fact that this study focussed on one of Europe’s most deprived areas^50^, our findings establish the foundation of the crucial role blue spaces could play in levelling inequalities in mental health. In addition, this raises the question of equity of access to natural environments in urban settings. For instance, while green spaces appear to have similar salutogenic effects on mental health^51^, access and geographical distribution of green spaces are strongly determined by area deprivation status^52–55^. In contrast, blue spaces are thought to be more evenly distributed socio-economically^31^. However, recent evidence has indicated that those better off are more likely to visit blue spaces than people with lower incomes^56^. Therefore, equitable use and access to blue space should be actively encouraged. Such outcomes can be achieved through the development and promotion of nature-based solutions that encourage opportunities to be physically active and provide restorative and therapeutic spaces for mental health^57^.

Secondly, this study illustrates the importance for health professionals to work closely with urban planners and engage in urban regeneration programmes to address mental health disorders in urban populations. Indeed, as per the Town and Country Planning Association^58^, inland waterways are recognised as catalysts for urban regeneration, which promote health and wellbeing. This policy also presents underperforming waterways as the result of the economic and social failure of adjacent neighbourhoods^58^. Building on current policy, this study provides evidence for the link between socio-economic deprivation and blue space benefits, and suggests that waterway regeneration efforts should primarily focus on the most deprived areas.

Thirdly, another important aspect of this study is that the driving force and incentive of the Glasgow Branch of the Forth and Clyde Canal regeneration effort was the creation of a Nature-Based Solution for climate adaptation. Although the impact on health was hypothesised, it was not taken into account directly. Therefore, it is evident that urban climate adaptation efforts can have significant mental health co-benefits. This is an important finding because health and climate change vulnerability tend to cluster^59^. Waterway regeneration efforts could therefore not only help towards climate adaptation but also lead to significant co-benefits by alleviating the consequences of socio-economic deprivation and, in turn, improving mental health.

To our knowledge, this is the first longitudinal study looking at the modifying role of blue space on the association between socio-economic deprivation and mental health, using objective clinical mental health measures. The uniqueness of this study also lies in the fact that the North Glasgow branch of the Forth and Clyde canal offers the opportunity to understand the impact of blue spaces in disadvantaged communities^60^.

A possible limitation of our study is that NHS records of GP visits and drug prescription records may not capture all diagnoses of mental health disorders. Studies have shown that people with mental health disorders and/or low socio-economic status often experience social discrimination and stigmatisation^61,62^. In healthcare, stigma raises a significant barrier for the most vulnerable and reduces access to diagnosis, treatment and successful health outcomes, while cost may also be a factor^63^. Therefore, the trend of blue space benefits for those living closer to the canal may reflect the consequences of stigmatisation towards people with mental disorders. However, the levels of usage of NHS services in our dataset were found to be stable across all distances and time periods. Even so, we did not have access to psychotherapy NHS data. It is therefore still possible that a number of people with mental health disorders may have not sought treatment and therefore effects may have been underestimated. Lastly, a certain limitation is that living within closer proximity to blue space does not fully reflect use of blue spaces^64^.

In conclusion, our findings indicate that living near urban blue space can alleviate the adverse impacts of socio-economic deprivation on mental health and in turn potentially reduce medication intake, especially for those most deprived. Our study suggests that increasing exposure to blue spaces through the development or regeneration of blue spaces could potentially be a powerful tool to reduce mental health inequalities in urban populations.

## Supplementary Information

Supplementary Information.

## Data Availability

Data for this study are available through the Greater Glasgow and Clyde NHS Safe Haven secure environment and access is codified by NHS ethics.

## Code availability

Codes can be provided by the corresponding author (MG) upon reasonable request.
