# Supplementary material for: A population-based retrospective study of the modifying effect of urban blue space on the impact of socioeconomic deprivation on mental health, 2009–2018
Source: Sci Rep. 2022 Jul 29;12:13040. doi: 10.1038/s41598-022-17089-z (PMC9338232; doi:10.1038/s41598-022-17089-z)
Supplement: Supplementary file 1 — Supplementary Information. [file 41598_2022_17089_MOESM1_ESM.docx]

A population-based retrospective study of the modifying effect of urban blue space on the impact of socioeconomic deprivation on mental health, 2009-2018

Michail Georgiou ^1*^, Zoë Tieges^1,4,5^, Gordon Morison^2^, Niamh Smith^1^, Sebastien Chastin^1,3^

^1^ School of Health and Life Sciences, Glasgow Caledonian University, 70 Cowcaddens Road,

Glasgow G4 0BA, Scotland, UK

^2^ School of Computing, Engineering and Built Environment, Glasgow Caledonian University, 70 Cowcaddens Road, Glasgow G4 0BA, Scotland, UK

^3^ Department of Movement and Sports, Ghent University, Watersportlaan 2, 9000 Ghent, Belgium.

^4^ Geriatric Medicine, Usher Institute, University of Edinburgh, 51 Little France Crescent, Edinburgh EH16 4SA, Scotland, UK.

^5^ SMART Technology Centre, School of Computing, Engineering and Built Environment, Glasgow Caledonian University, 70 Cowcaddens Road, Glasgow G4 0BA, Scotland, UK

***** Correspondence to: Michail Georgiou (M.G); [Michail.georgiou@gcu.ac.uk](mailto:Michail.georgiou@gcu.ac.uk); ORCID 0000-0002-7652-8683

Supplementary Material

**Drugs Included**

Table S1: List of drugs included in the analysis

| **Drug Name** |
| --- |
| Mirtazapine |
| Amitriptyline |
| Trazodone Hydrochloride |
| Venlafaxine |
| Nortriptyline |
| Lofepramine |
| Imipramine Hydrochloride |
| Dosulepin Hydrochloride |
| Reboxetine |
| Flupentixol |
| Duloxetine |
| Chlomipramine Hydrochloride |
| Doxepin |
| Trimipramine |
| Agomelatine |
| Mianserin Hydrochloride |
| Tryptophan |
| Vortioxetine Hydrobromide |
| Amitriptyline Hydrochloride with perphenazine |

**Hazard Ratios (main analysis)**

Table S2: Hazard Ratios of the association between mental health related drug prescription and distance to canal

| **Model** | **Variable** | **HR** | **Lower Bound** | **Upper Bound** |
| --- | --- | --- | --- | --- |
| Base Model | Age | 1.02 | 1.02 | 1.02 |
| Base Model | Sex (Males) | 0.69 | 0.67 | 0.71 |
| Base Model | Comorbidities | 1.71 | 1.64 | 1.77 |
| Base Model | SIMD T1 | 2.54 | 2.45 | 2.63 |
| Base Model | SIMD T2 | 1.70 | 1.63 | 1.76 |
| Model with Distance to Blue Space | Age | 1.02 | 1.02 | 1.02 |
| Model with Distance to Blue Space | Sex (Males) | 0.69 | 0.67 | 0.71 |
| Model with Distance to Blue Space | Comorbidities | 1.71 | 1.64 | 1.77 |
| Model with Distance to Blue Space | SIMD T1 | 2.48 | 2.39 | 2.57 |
| Model with Distance to Blue Space | SIMD T2 | 1.66 | 1.60 | 1.72 |

**Sensitivity Analysis**

Figure S1: Sensitivity analysis forest plot. *Distance to blue space variable shown as distance to BS.


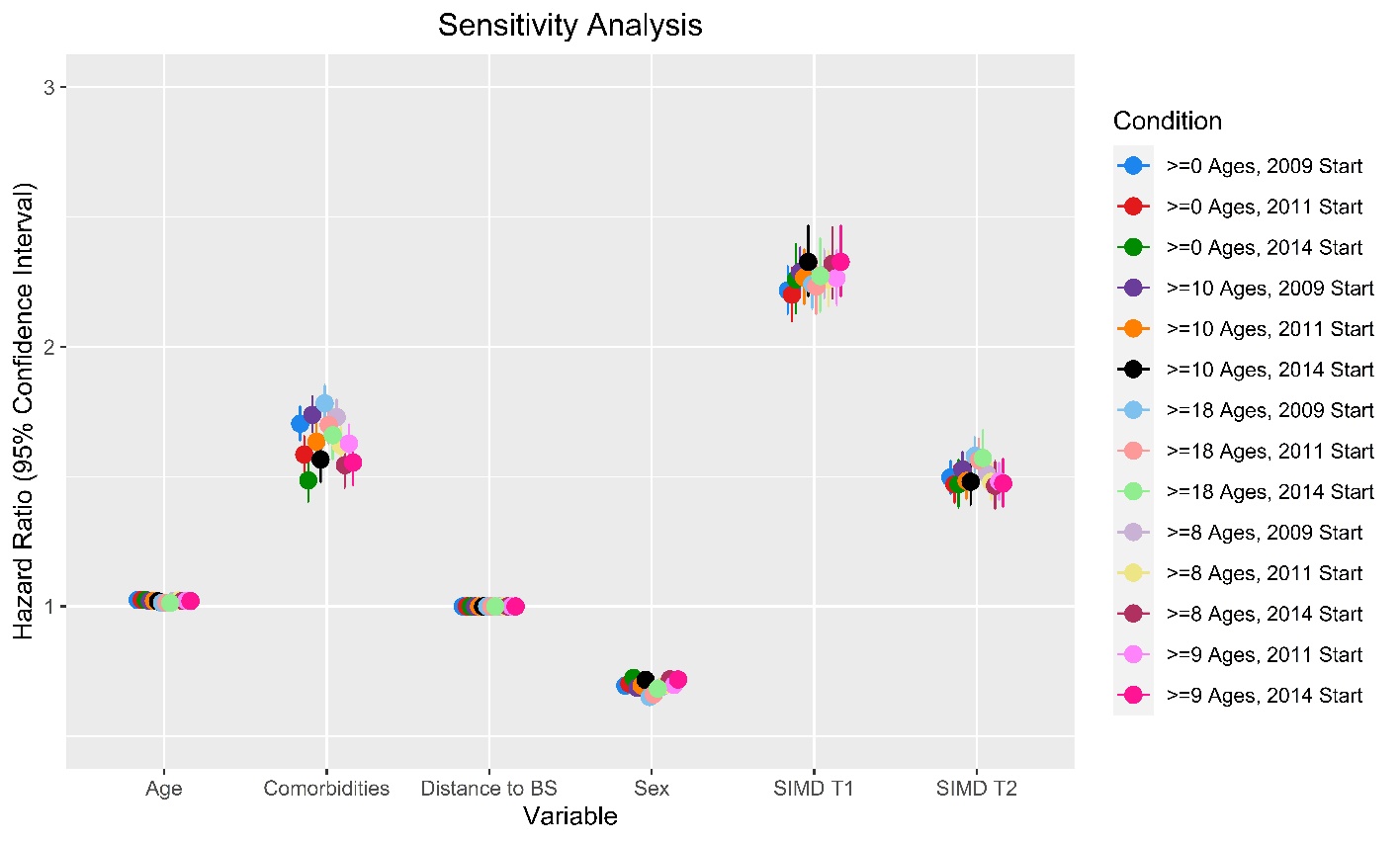


Table S3: Sensitivity analysis hazard ratios for each model and variable

| **Condition** | **Variable** | **HR** | **Lower Bound** | **Upper Bound** |
| --- | --- | --- | --- | --- |
| >=0 Ages, 2009 Start | Distance to Blue Space | 1.00 | 1.00 | 1.00 |
| >=0 Ages, 2009 Start | Age | 1.02 | 1.02 | 1.02 |
| >=0 Ages, 2009 Start | Sex | 0.69 | 0.68 | 0.71 |
| >=0 Ages, 2009 Start | Comorbidities | 1.70 | 1.64 | 1.77 |
| >=0 Ages, 2009 Start | SIMD T1 | 2.22 | 2.13 | 2.31 |
| >=0 Ages, 2009 Start | SIMD T2 | 1.50 | 1.43 | 1.56 |
| >=0 Ages, 2011 Start | Distance to Blue Space | 1.00 | 1.00 | 1.00 |
| >=0 Ages, 2011 Start | Age | 1.02 | 1.02 | 1.03 |
| >=0 Ages, 2011 Start | Sex | 0.70 | 0.68 | 0.72 |
| >=0 Ages, 2011 Start | Comorbidities | 1.59 | 1.52 | 1.66 |
| >=0 Ages, 2011 Start | SIMD T1 | 2.20 | 2.10 | 2.31 |
| >=0 Ages, 2011 Start | SIMD T2 | 1.47 | 1.40 | 1.54 |
| >=0 Ages, 2014 Start | Distance to Blue Space | 1.00 | 1.00 | 1.00 |
| >=0 Ages, 2014 Start | Age | 1.02 | 1.02 | 1.03 |
| >=0 Ages, 2014 Start | Sex | 0.72 | 0.70 | 0.75 |
| >=0 Ages, 2014 Start | Comorbidities | 1.49 | 1.40 | 1.57 |
| >=0 Ages, 2014 Start | SIMD T1 | 2.26 | 2.13 | 2.40 |
| >=0 Ages, 2014 Start | SIMD T2 | 1.47 | 1.38 | 1.57 |
| >=8 Ages, 2009 Start | Distance to Blue Space | 1.00 | 1.00 | 1.00 |
| >=8 Ages, 2009 Start | Age | 1.02 | 1.02 | 1.02 |
| >=8 Ages, 2009 Start | Sex | 0.69 | 0.67 | 0.71 |
| >=8 Ages, 2009 Start | Comorbidities | 1.73 | 1.66 | 1.80 |
| >=8 Ages, 2009 Start | SIMD T1 | 2.28 | 2.19 | 2.37 |
| >=8 Ages, 2009 Start | SIMD T2 | 1.51 | 1.45 | 1.58 |
| >=8 Ages, 2011 Start | Distance to Blue Space | 1.00 | 1.00 | 1.00 |
| >=8 Ages, 2011 Start | Age | 1.02 | 1.02 | 1.02 |
| >=8 Ages, 2011 Start | Sex | 0.70 | 0.68 | 0.72 |
| >=8 Ages, 2011 Start | Comorbidities | 1.62 | 1.55 | 1.69 |
| >=8 Ages, 2011 Start | SIMD T1 | 2.26 | 2.16 | 2.37 |
| >=8 Ages, 2011 Start | SIMD T2 | 1.48 | 1.41 | 1.55 |
| >=8 Ages, 2014 Start | Distance to Blue Space | 1.00 | 1.00 | 1.00 |
| >=8 Ages, 2014 Start | Age | 1.02 | 1.02 | 1.02 |
| >=8 Ages, 2014 Start | Sex | 0.72 | 0.69 | 0.75 |
| >=8 Ages, 2014 Start | Comorbidities | 1.54 | 1.46 | 1.64 |
| >=8 Ages, 2014 Start | SIMD T1 | 2.32 | 2.19 | 2.46 |
| >=8 Ages, 2014 Start | SIMD T2 | 1.46 | 1.38 | 1.56 |
| >=9 Ages, 2011 Start | Distance to Blue Space | 1.00 | 1.00 | 1.00 |
| >=9 Ages, 2011 Start | Age | 1.02 | 1.02 | 1.02 |
| >=9 Ages, 2011 Start | Sex | 0.70 | 0.68 | 0.72 |
| >=9 Ages, 2011 Start | Comorbidities | 1.63 | 1.56 | 1.70 |
| >=9 Ages, 2011 Start | SIMD T1 | 2.26 | 2.16 | 2.37 |
| >=9 Ages, 2011 Start | SIMD T2 | 1.48 | 1.41 | 1.55 |
| >=9 Ages, 2014 Start | Distance to Blue Space | 1.00 | 1.00 | 1.00 |
| >=9 Ages, 2014 Start | Age | 1.02 | 1.02 | 1.02 |
| >=9 Ages, 2014 Start | Sex | 0.72 | 0.69 | 0.75 |
| >=9 Ages, 2014 Start | Comorbidities | 1.55 | 1.47 | 1.65 |
| >=9 Ages, 2014 Start | SIMD T1 | 2.33 | 2.19 | 2.47 |
| >=9 Ages, 2014 Start | SIMD T2 | 1.47 | 1.38 | 1.57 |
| >=10 Ages, 2009 Start | Distance to Blue Space | 1.00 | 1.00 | 1.00 |
| >=10 Ages, 2009 Start | Age | 1.02 | 1.02 | 1.02 |
| >=10 Ages, 2009 Start | Sex | 0.69 | 0.67 | 0.71 |
| >=10 Ages, 2009 Start | Comorbidities | 1.74 | 1.67 | 1.81 |
| >=10 Ages, 2009 Start | SIMD T1 | 2.29 | 2.19 | 2.38 |
| >=10 Ages, 2009 Start | SIMD T2 | 1.53 | 1.46 | 1.59 |
| >=10 Ages, 2011 Start | Distance to Blue Space | 1.00 | 1.00 | 1.00 |
| >=10 Ages, 2011 Start | Age | 1.02 | 1.02 | 1.02 |
| >=10 Ages, 2011 Start | Sex | 0.69 | 0.67 | 0.72 |
| >=10 Ages, 2011 Start | Comorbidities | 1.63 | 1.56 | 1.71 |
| >=10 Ages, 2011 Start | SIMD T1 | 2.27 | 2.16 | 2.37 |
| >=10 Ages, 2011 Start | SIMD T2 | 1.48 | 1.41 | 1.56 |
| >=10 Ages, 2014 Start | Distance to Blue Space | 1.00 | 1.00 | 1.00 |
| >=10 Ages, 2014 Start | Age | 1.02 | 1.02 | 1.02 |
| >=10 Ages, 2014 Start | Sex | 0.72 | 0.69 | 0.75 |
| >=10 Ages, 2014 Start | Comorbidities | 1.57 | 1.48 | 1.66 |
| >=10 Ages, 2014 Start | SIMD T1 | 2.33 | 2.19 | 2.47 |
| >=10 Ages, 2014 Start | SIMD T2 | 1.48 | 1.39 | 1.58 |
| >=18 Ages, 2009 Start | Distance to Blue Space | 1.00 | 1.00 | 1.00 |
| >=18 Ages, 2009 Start | Age | 1.01 | 1.01 | 1.01 |
| >=18 Ages, 2009 Start | Sex | 0.65 | 0.63 | 0.67 |
| >=18 Ages, 2009 Start | Comorbidities | 1.78 | 1.71 | 1.85 |
| >=18 Ages, 2009 Start | SIMD T1 | 2.24 | 2.15 | 2.34 |
| >=18 Ages, 2009 Start | SIMD T2 | 1.58 | 1.51 | 1.65 |
| >=18 Ages, 2011 Start | Distance to Blue Space | 1.00 | 1.00 | 1.00 |
| >=18 Ages, 2011 Start | Age | 1.01 | 1.01 | 1.02 |
| >=18 Ages, 2011 Start | Sex | 0.66 | 0.64 | 0.68 |
| >=18 Ages, 2011 Start | Comorbidities | 1.70 | 1.63 | 1.78 |
| >=18 Ages, 2011 Start | SIMD T1 | 2.23 | 2.13 | 2.34 |
| >=18 Ages, 2011 Start | SIMD T2 | 1.56 | 1.49 | 1.64 |
| >=18 Ages, 2014 Start | Distance to Blue Space | 1.00 | 1.00 | 1.00 |
| >=18 Ages, 2014 Start | Age | 1.01 | 1.01 | 1.01 |
| >=18 Ages, 2014 Start | Sex | 0.68 | 0.65 | 0.71 |
| >=18 Ages, 2014 Start | Comorbidities | 1.66 | 1.57 | 1.76 |
| >=18 Ages, 2014 Start | SIMD T1 | 2.27 | 2.13 | 2.42 |
| >=18 Ages, 2014 Start | SIMD T2 | 1.57 | 1.47 | 1.68 |
